# The Expression of Ribonucleotide Reductase M2 in the Carcinogenesis of Uterine Cervix and Its Relationship with Clinicopathological Characteristics and Prognosis of Cancer Patients

**DOI:** 10.1371/journal.pone.0091644

**Published:** 2014-03-17

**Authors:** Ying-Fang Su, Tzu-Fan Wu, Jiunn-Liang Ko, Hsiu-Ting Tsai, Yi-Torng Tee, Ming-Hsien Chien, Chi-Hung Chou, Wea-Lung Lin, Hui-Ying Low, Ming-Yung Chou, Shun-Fa Yang, Po-Hui Wang

**Affiliations:** 1 Institute of Medicine, Chung Shan Medical University, Taichung, Taiwan; 2 School of Dentistry, Chung Shan Medical University, Taichung, Taiwan; 3 Department of Stomatology, Chung Shan Medical University Hospital, Taichung, Taiwan; 4 School of Nursing, Chung Shan Medical University, Taichung, Taiwan; 5 Department of Nursing, Chung Shan Medical University Hospital, Taichung, Taiwan; 6 School of Medicine, Chung Shan Medical University, Taichung, Taiwan; 7 Department of Obstetrics and Gynecology, Chung Shan Medical University Hospital, Taichung, Taiwan; 8 Graduate Institute of Clinical Medicine, College of Medicine, Taipei Medical University, Taipei, Taiwan; 9 Wan Fan Hospital, Taipei Medical University, Taipei, Taiwan; 10 Division of Cardiology, Department of Internal Medicine, Yuan-Sheng Hospital and Changhua Christian Hospital, Yuanlin Branch, Yuanlin, Taiwan; 11 Department of Pathology, Chung Shan Medical University Hospital, Taichung, Taiwan; China Medical University, Taiwan

## Abstract

**Background:**

To investigate the implication of ribonucleotide reductase M2 (RRM2) in the carcinogenesis of uterine cervix and its relationship with clinicopathological characteristics and prognosis of cancer patients.

**Methodology and Principal Findings:**

The impact of RRM2 on cell viability was investigated in SiHa cervical cancer cells after RRM2 knockdown and the addition of cisplatin, which induces inter- and intra-strand DNA crosslinks. RRM2 immunoreactivity was evaluated by semi-quantitative H score among 29 normal, 30 low-grade dysplasia, 30 high-grade dysplasia and 103 invasive cancer tissue specimens of the uterine cervix, using tissue microarrays. RRM2 was then correlated with the clinicopathological variables of cervical cancer and patient survival. A greater toxic effect on cell viability using cisplatin was reflected by the greater reduction in RRM2 protein expression in SiHa cells. The RRM2 expression in cancer tissues was higher than that in high-grade dysplasia, low-grade dysplasia or normal cervical tissues. RRM2 upregulation was correlated with deep stromal invasion, large tumors and parametrial invasion and predicted poor survival.

**Conclusions:**

RRM2 is a new molecular marker for the diagnosis and clinical outcomes of cervical cancer. It is involved in cervical carcinogenesis and predicts poor survival, and may be a potential therapeutic target including in cisplatin treatment.

## Introduction

Ribonucleotide reductase (RNR) is an enzyme that catalyzes the formation of deoxyribonucleotides from ribonucleotides [1]. Deoxyribonucleotides in turn are used in the synthesis of deoxyribonucleic acid (DNA) and DNA repair [1], [2]. RNR consists of two protein subunits referred to as large R1 (termed as M1 in humans) and small R2 [3]. R2 is classified as RRM2 (ribonucleotide reductase M2) and p53R2 subunits which form an active heterodimeric tetramer in humans [4], [5]. RNR has been reported to be correlated with cell proliferation and differentiation [6], [7], and RNR activity needs to be coordinated with cell cycle progression to preserve the balance between dNTP production and DNA replication. The concentrations of R1 protein are relatively stable throughout the cell cycle and are always in excess of RRM2 [8]. Therefore, the cell cycle-dependent activity of RNR is regulated by RRM2 levels [8].

The destruction of cancer cells by cisplatin requires the binding of the drug to DNA and the formation of platinum-DNA adducts, which may establish inter- and intra-strand DNA crosslinks, thereby inhibiting DNA replication [9]. The excision repair cross-complementation group 1 (ERCC1) enzyme has been reported to play an important role in the repair of inter-strand crosslinks in DNA [10]–[12]. In addition, R2 expression is cell cycle-dependent, with the highest level concurrent with DNA replication [4], [8], suggesting that RRM2 fuels DNA repair. Our preliminary experiment showed that SiHa cervical cancer cells, which have a higher RRM2 protein content, exhibit higher cell viability than Caski cervical cancer cells, which have a lower RRM2 protein content, based on MTT [3-(4,5-cimethylthiazol-2-yl)- 2,5-diphenyl tetrazolium bromide] assay after the treatment of cisplatin. The expression levels of RRM2 protein of SiHa cervical cancers were 2.3–3.6 folds more than those of Caski cervical cancers. After 48 hours culture with the addition of 5 µM cisplatin, the cell viability of SiHa cells was about 80% of original SiHs cells without cisplatin addition, whereas, the cell viability of Caski cells was reduced to 51% (SiHa vs. Caski: 80% vs. 51%, *p*<0.001). This implies that cervical cancer cells with elevated RRM2 expression have better cell viability.

To the best of our knowledge, the involvement of RRM2 in cervical carcinogenesis and patient survival has not been reported previously. Therefore, the objectives of this study were to investigate the clinical implication of RRM2 in cervical cancer. We hypothesized that RRM2 is correlated with the progression of cancer of uterine cervix. We detected whether cancer cell viability was reduced after the addition of cisplatin using MTT assay if the RRM2 gene was knocked down in cervical cancer cells. If RRM2 could affect cancer cells proliferation, we further examined RRM2 immunoreactivity in normal, low-grade and high-grade dysplasia tissues and invasive cancer tissue specimens of the uterine cervix and defined the correlation of RRM2 expression with cervical cancer carcinogenesis. Moreover, we associated the expression of RRM2 with clinicopathological variables of patients with cervical cancer, and investigated the relationship of its expression with cancer recurrence and patient survival.

## Materials and Methods

### Cell culture

SiHa and human embryonic kidney 293T cell lines were obtained from the American Type Tissue Culture Collection (ATCC; Rockville, MD, USA). SiHa and 293T cell lines were grown in Dulbecco's Modified Eagle Medium (DMEM; Gibco, Grand Island, NY) supplemented with 10% heat-inactivated fetal bovine serum (FBS; Gibco, Grand Island, NY).

### Lentivirus production and transduction

The 293T cells were transfected with 5 µg short hairpin RNA (shRNA) plasmid, 4 µg pCMVDR8.91 and 0.4 µg pMD.G by jetPEI DNA transfection reagent based on the manufacturer's protocol (PolyPlus-transfection, 101-10; Strasbourg, France). Predesigned shRNA and target sequences were purchased from the National RNAi Core Facility as follows: shRRM2 #354 (TRCN0000286354): 5′- CGG AGG AGA GAG TAA GAG AAA -3′; shRRM2 #962 (TRCN0000038962): 5′- GCT CAA GAA ACG AGG ACT GAT-3′ and shLuc (TRCN0000072246): CAA ATC ACA GAA TCG TCG TAT. After 24 hours of culture, the SiHa cervical cancer cells were infected with recombinant lentivirus vectors at a multiplicity of infection (MOI) of 1. The next day, the medium was removed and the cells were selected by 2 µg/ml puromycin (Sigma, P8833, St. Louis, MO). The SiHa shRRM2 #354 and SiHa shRRM2 #962 cell lines were established.

### Western blot analysis

An equivalent amount of total protein was processed by 12% SDS-PAGE and electroblotted onto Hybond ECL PVDF membranes, incubated with primary antibodies against human RRM2 (GTX103193, GeneTex, Inc.; Irvine, CA 92606 USA) and β-actin (A5441, Sigma, St. Louis, MO).

### MTT [3- (4,5-cimethylthiazol-2-yl)-2,5-diphenyl tetrazolium bromide] assay

SiHa shRRM2 #354, SiHa shRRM2 #962 and SiHa shLuc cells were seeded onto a 96-well microculture plate at 5000 cells/well and allowed to attach overnight. The next day, the cells were exposed to different concentrations (0, 25, 50 µM) of cisplatin in DMEM medium supplemented with 10% FBS and incubated for 48 hours. The medium was replaced with fresh medium containing MTT (0.2 mg/ml) and the plates were incubated for another 3 hours. The medium was then removed and dimethyl sulfoxide (DMSO) was added to dissolve the MTT formazan crystals. Absorbance of the color was measured at 570 nm, and cell viability was calculated as the percentage of viable cells in the total population.

### Study subjects and immunohistochemical expression of RRM2 in cancer, high-grade and low-grade dysplasia and normal tissues using tissue microarrays

We constructed two formalin-fixed, paraffin-embedded tissue microarrays (MaxArray tissue cores, Zymed, South San Francisco, California), consisting of 29 normal, 30 low-grade dysplasia, 30 high-grade dysplasia and 103 invasive cancer tissue specimens of the uterine cervix. These tissue specimens were collected from histopathological paraffin blocks of the Pathology Departments in Chung Shan Medical University. A total of 103 patients with cervical cancer were recruited consecutively between March 1999 and March 2006. They were staged according to the 2009 International Federation of Gynecology and Obstetrics (FIGO) Classification [13] and received standard treatment protocols at the Department of Obstetrics and Gynecology, Chung Shan Medical University Hospital, Taiwan. Thirty patients with high-grade dysplasia had received large loop excisions of the transformation zone, total abdominal hysterectomy (TAH), or total vaginal hysterectomy (TVH). The pathological diagnosis of these samples revealed moderate, severe dysplasia, or squamous cell carcinoma (SCC) in situ. Thirty patients had low-grade dysplasia for which they received colposcopy-directed punch biopsies, all found to have mild dysplasia by pathological studies. Twenty-nine normal tissue specimens of the uterine cervix were collected from patients receiving TAH or TVH for benign uterine disease without cervical lesions. Tissue microarray sections (MaxArray with 96 tissue cores in each tissue microarray, Zymed Laboratories Inc.) were incubated with anti-RRM2 antibodies (ab57653, Abcam Inc. MA, USA). A semi-quantitative H score of RRM2 immunoreactivity was determined by multiplying the proportional score of stained cells by their immunoreactivity intensity [14], [15]. The primary clinical endpoint was to correlate the expression of RRM2 with the survival of patients with cervical cancer. This study was approved by the Chung Shan Medical University Hospital Institutional Review Board (CSMUH IRB; CS12218 and CS12242) and informed consent was obtained from each patient. We followed the REMARK recommendations for tumor markers in this study [16].

### Statistical analysis

The Kruskal-Wallis H method was used to analyze the differences in RRM2 expression among the cervical cancer, high-grade dysplasia, low-grade dysplasia and normal tissue samples. The Mann-Whitney test was used for post-hoc analysis. In response to multiple analyses, the Hommel method was used to adjust the *p* values using WinPepi software, version 10.0.

We associated the expressions of RRM2 with clinicopathological characteristics of the patients with cervical cancer using chi-square or Fisher's exact tests. *P* values and odds ratios were analyzed by WinPepi Software, version 10.0. Kaplan-Meier curves were plotted for the cervical cancer patients based on the RRM2 expression for the probability of recurrence or overall survival between primary surgery and death or recurrence or the end of the study (May 31, 2012). Mean (median) survival times and 5-year survival rates were estimated by the Kaplan-Meier product limit method. Multivariate and univariate Cox regression models were used to assess the prognostic value of biological and clinical parameters with or without adjustment for RRM2 expression and clinicopathological variables. The statistical analyses were performed using SPSS statistical software (version 11.0; SPSS, Inc., Chicago, IL). All statistical tests were two-sided and a *p* value of less than 0.05 was considered to be statistically significant.

## Results

**Figure 1:**
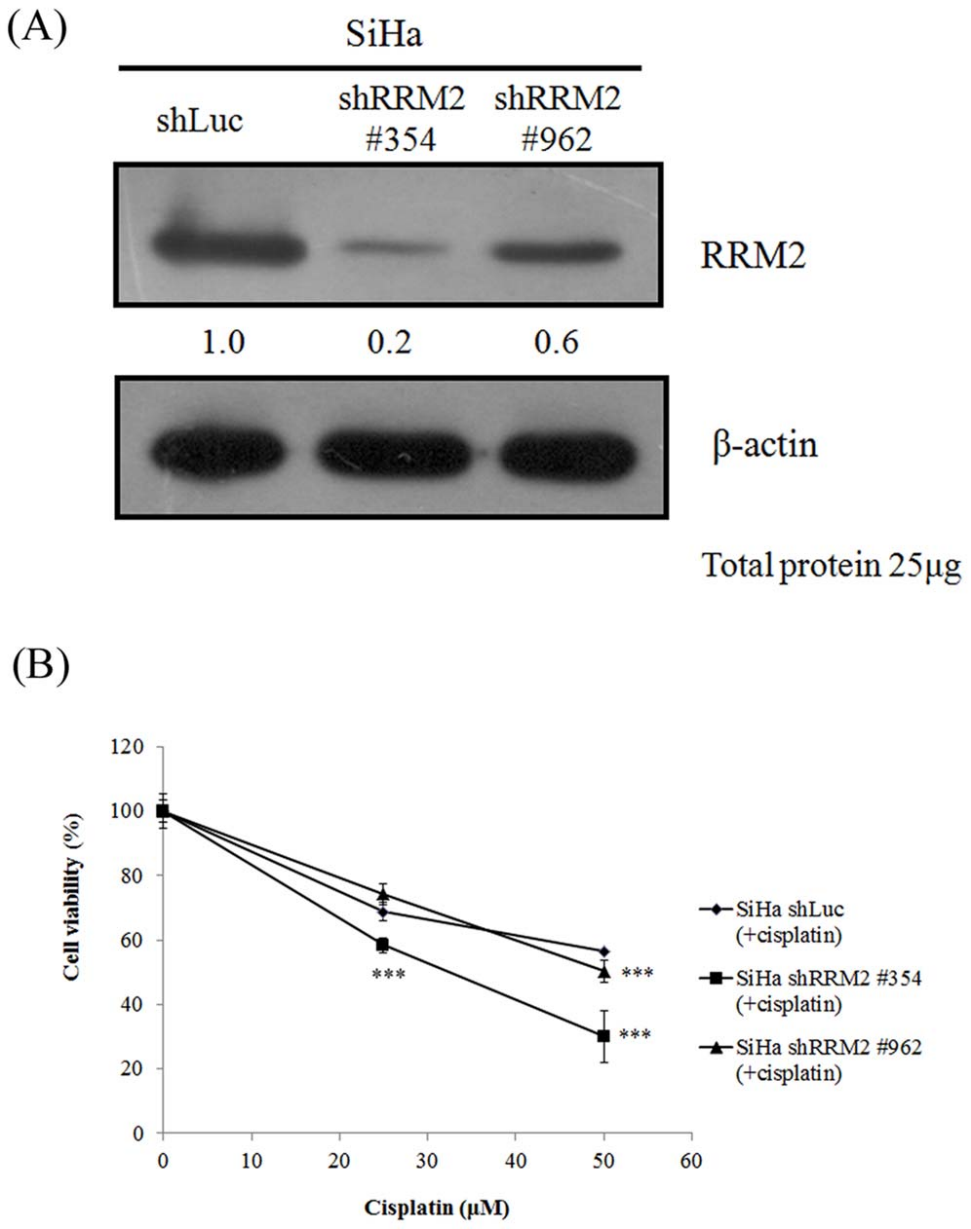
RRM2 protein expressions in the SiHa cancer cells of the uterine cervix with shRRM2 #354 or #962 and their correlations with cell viability. (A) Protein levels of RRM2 were detected in the SiHa cervical cancer cells with shRRM2 #354 or #962 using Western blotting. β-actin was used as the internal control. The relative expressions of RRM2/β-actin are showed. The effect of RRM2 gene knockdown was stronger in the SiHa cells with shRRM2 #354 compared to those with shRRM2 #962. (B) A greater toxic effect on cell viability using cisplatin was found in the SiHa cells with shRRM2 #354 than in those with shRRM2 #962 compared to the control SiHa shLuc cells, in which shLuc was transfected into SiHa cells. Cell viabilities were detected using 3-(4,5-cimethylthiazol-2-yl)-2,5- diphenyl tetrazolium bromide assay. RRM2, ribonucleotide reductase M2; ***p<0.001.

When the RRM2 gene was knocked down in the SiHa cervical cancer cells using shRRM2 #354 and RRM2 #962, the level of RRM2 protein was reduced more significantly in the SiHa shRRM2 #354 than in the #962 cell lines (Figure 1A). When 25 µM of cisplatin was added to the SiHa shRRM2 #962 and #354 cell lines, the cell viability was not reduced in the #962 cells but was significantly reduced in the #354 cells (*p*<0.001) compared to the control SiHa shLuc cells, in which the control vector shLuc was transfected into the SiHa cervical cancer cells (Figure 1B). When 50 µM of cisplatin was added to the SiHa shRRM2 #962 and #354 cell lines, the cell viability was reduced in both #962 (*p*<0.001) and #354 cells (*p*<0.001) compared to the control SiHa shLuc cells (Figure 1B).

**Figure 2:**
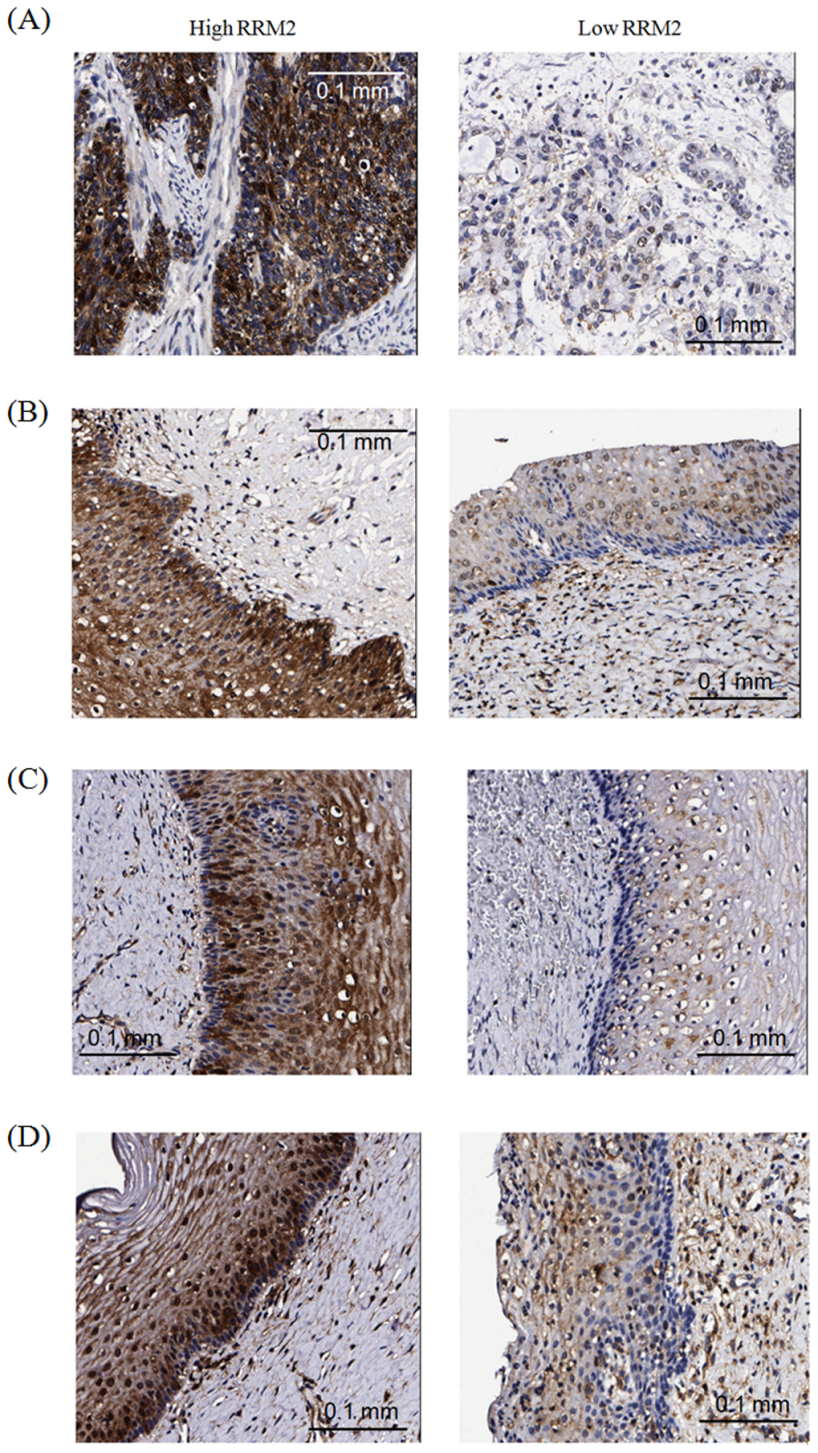
Immunoreactivity of invasive, high-grade dysplasia, low-grade dysplasia and normal tissues of the uterine cervix. (A) High RRM2 immunostaining (left) and low RRM2 immunostaining (right) in cervical cancer tissues. (B) High RRM2 immunostaining (left) and low RRM2 immunostaining (right) in cervical high-grade dysplasia. (C) High RRM2 immunostaining (left) and low RRM2 immunostaining (right) in cervical low-grade dysplasia. (D) High RRM2 immunostaining (left) and low RRM2 immunostaining (right) in normal cervical tissues. Magnification: 200×; RRM2, ribonucleotide reductase M2.

The staining of RRM2 was nuclear and the expression pattern of RRM2 protein in tumor tissues was in tumor cores in cervical cancer tissue microarrays. (Figure 2). The median value of all H scores of the tumor cells in the 103 cervical cancer cores was 1.0. The median H scores of the 29 normal, 30 low-grade dysplasia and 30 high-grade dysplasia tissues were 0.5, 0.5 and 0.5, respectively. The RRM2 expression was significantly different among the cervical cancer, high-grade dysplasia, low-grade dysplasia and normal tissues (*p*<0.001, Kruskal-Wallis H method). The RRM2 expression in the cancer tissues was higher than that in high-grade dysplasia (median H score: 1.0 vs. 0.5; Hommel's adjusted *p*<0.001), low-grade dysplasia (median H score: 1.0 vs. 0.5; Hommel's adjusted *p* = 0.002) or normal (median H score: 1.0 vs. 0.5; Hommel's adjusted *p*<0.001) tissues. When the H scores of RRM2 in the cervical cancer tissues were a median value of 1 or more, their immunohistochemical expressions were regarded as being high (positive); otherwise, they were regarded as being low (negative).

The mean age of the patients with cervical cancer was 51.4±11.8 years. The patients with invasive cancer of the uterine cervix were clinically staged based on the 2009 FIGO Classification, and 68 patients were staged I, 32 staged II, 2 staged III and 1 staged IV. The median follow-up time after surgery was 61.0 months and the mean survival time was 103 months. The 5-year survival rate was 80.6%. Two patients were lost to follow-up, 25 patients died and 16 patients relapsed. Staining for RRM2 was stronger in the older patients [>50 years; *p* = 0.004, odds ratio (OR): 4.97], those with advanced stage disease (≥stage II; *p* = 0027; OR: 4.01), deep stromal invasion (*p*<0.001; OR: 12.46), large tumors (*p* = 0.001; OR: >2.62), moderate or poor cell differentiation (*p*<0.001; OR: 15.98), parametrial invasion (*p* = 0.022; OR: 7.97), and it tended to be stronger in the patients with lymph node metastasis (*p* = 0.066; OR: 6.10) (Table 1).

**Table 1:** The correlation of ribonucleotide reductase M2 (RRM2) immunoreactivity in 103 cancer tissue cores with clinicopathological variables in the cervical cancer patients.

Clinicopathological variables^a^	RRM2^b^		*p* value	OR and 95%CI
	(+)	(−)		
Age (years)				
≤50	38	18	0.004	1.00
>50	42	4		4.97 (1.44–21.69)
Stage				
I	49	19	0.027	1.00
others	31	3		4.01 (1.04–22.61)
Pathologic type				
squamous cell carcinoma	67	16	0.362	1.00
adenocarcinoma	14	6		0.56 (0.17–2.06)
Depth of stromal invasion				
≤1/2 stromal depth	26	18	<0.001	1.00
>1/2 stromal depth	54	3		12.46 (3.15–70.02)
Tumor diameter				
≤4 cm	50	21	0.001	1.00
>4 cm	28	0		∞ (2.62-∞)
Tumor grade				
well	6	11	<0.001	1.00
moderate or poor	61	7		15.98 (3.83–68.66)
Parametrial invasion				
no invasion	58	21	0.022	1.00
Invasion	22	1		7.97 (1.12–344.14)
Vaginal invasion				
no invasion	66	21	0.181	1.00
Invasion	14	1		4.45 (0.60–197.18)
Pelvic lymph node metastasis				
negative	62	21	0.066	1.00
positive	18	1		6.10 (0.84–265.92)

Statistical analysis: Chi-square or Fisher's exact tests.

a Some clinicopathological data could not be collected from the patients with cervical cancer due to incomplete medical charts or records.

b (+): positive immunoreactivity; (−): negative immunoreactivity.

Semiquantitative H score of RRM2 immunoreactivity was calculated by multiplying the proportional score of stained cells and their immunoreactivity intensity.

The median value of all H scores of tumor cells in the 103 cervical cancer cores was determined as the cutoff point for separating RRM2 positive tissue cores from RRM2 negative tissue cores.

OR: odds ratio; CI: confidence interval; RRM2, ribonucleotide reductase M2.

Univariate analysis revealed that the patients with cancer tissues exhibiting positive RRM2 immunoreactivity had poorer survival than those with cancer tissues exhibiting negative RRM2 immunoreactivity [5-year survival rate: 76.4 vs. 95.5%; hazard ratio (HR): 7.94, 95% confidence interval (CI): 1.07–58.82; *p* = 0.016] (Table 2). Other significant factors for poor survival included deep stromal invasion, large tumor diameter, parametrial invasion and pelvic lymph node metastasis (Table 2). However, multivariate analysis revealed that poor survival was only found in the patients whose cancer tissues exhibited deep stromal invasion (HR: 6.25, 95% CI: 1.43–27.03, *p* = 0.015) (Table 2).

**Table 2:** Univariate and multivariate analyses for the correlation of clinicopathological variables and ribonucleotide reductase M2 (RRM2) with survival.

	Case number	Mean (median) survival month	5-year survival rate (%)	Hazard ratio	95% confidence interval	*p* value
**Univariate analysis**						
Age (years old)^a^						
≤50	56	109 (133)	79.1	1	Reference	0.25
>50	45	90 (110)	81.7	1.60	0.72–3.57	
Stage^a^						
I	67	107 (133)	84.8	1	Reference	0.26
others	34	88 (119)	72.5	1.58	0.71–3.54	
Pathologic type^a^						
squamous cell carcinoma	81	104 (132)	83.8	1	Reference	0.32
adenocarcinoma	20	95 (133)	67.1	1.59	0.63–4.00	
Depth of stromal invasion^a^						
≤1/2 depth	43	124 (133)	94.9	1	Reference	0.0004
>1/2 depth	57	82 (110)	70.1	6.90	2.03–23.26	
Tumor diameter^a^						
≤4 cm	70	111 (133)	89.1	1	Reference	0.013
>4 cm	27	74 (105)	63.6	2.84	1.20–6.76	
Tumor grade^a^						
well	17	126 (133)	93.8	1	Reference	0.072
moderate and poor	68	92 (115)	77.5	5.21	0.69–38.46	
Parametrial invasion^a^						
no invasion	78	109 (133)	85.7	1	Reference	0.0091
invasion	23	72 (109)	64.2	2.94	1.25–6.90	
Vagina invasion^a^						
no invasion	86	104 (133)	80.8	1	Reference	0.57
invasion	15	89 (110)	79.4	1.32	0.49–3.56	
Pelvic lymph node metastasis^a^						
negative	82	107 (133)	84.3	1	Reference	0.037
positive	19	78 (119)	64.1	2.39	1.03–5.56	
RRM2^a^						
negative	22	126 (132)	95.5	1	Reference	0.016
positive	79	96 (115)	76.4	7.94	1.07–58.82	
**Multivariate analysis**						
Depth of stromal invasion^a^						
≤1/2 depth	43	124 (133)	94.9	1	Reference	0.015
>1/2 depth	57	82 (110)	70.1	6.25	1.43–27.03	

Statistical analysis: Kaplan-Meier products limit method and multivariate and univariate Cox regression models.

a Some clinicopathological data could not be collected from the patients with cervical cancer due to incomplete medical charts or records.

**Figure 3:**
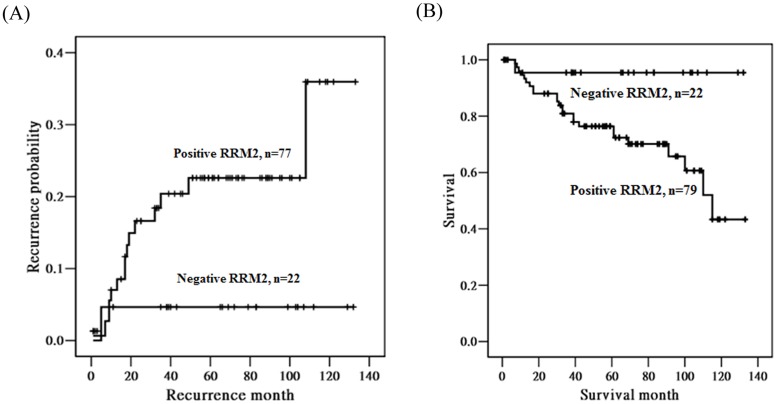
Kaplan-Meier curves for the probability of recurrence and overall survival in patients with cancer of the uterine cervix according to RRM2 immunoreactivity of cancer tissue cores. (A) Patients with positive RRM2 tended to have a higher probability of recurrence (N = 99, *p* = 0.094), compared with the patients with negative RRM2. (B) Patients with positive RRM2 had significantly lower survival (N = 101, *p* = 0.016), compared with the patients with negative RRM2. RRM2, ribonucleotide reductase M2.

Kaplan-Meier curves were plotted for the probability of recurrence and survival according to the expressions of RRM2 (Figure 3). We found that the patients with a positive RRM2 expression tended to have a higher probability of recurrence (*p* = 0.094) (Figure 3A). Furthermore, the patients with a positive RRM2 expression had a significantly poorer survival than those with a negative RRM2 expression (*p* = 0.016; Figure 3B).

Finally, we observed that the cervical patients receiving cisplatin-related treatment whose cancer tissues exhibited positive RRM2 immunoreactivity had a worse prognosis. Thirty-one patients with cervical cancer received concurrent chemoradiotherapy (CCRT) using cisplatin or adjuvant chemotherapy containing cisplatin. Of 28 patients who had cancer tissues positive for RRM2, 14 had recurrence or expired. However, in 3 patients who had cancer tissues negative for RRM2, none of them showed recurrence or expired.

## Discussion

In this study, we found that the cytotoxic effects of cisplatin were increased in cells with a greater reduction of RRM2 expression (shRRM2#354) as compared with cells with less reduction of RRM2 expression (shRRM2 #962). Because RRM2 may fuel DNA repair, and because the cell cycle-dependent activity of RNR is regulated by RRM2 levels [8], RRM2 is associated with cell proliferation. Therefore, if the RRM2 gene is knocked down in SiHa cervical cancer cells, cell proliferation may be affected. This is supported by the finding that knockdown of RRM2 protein markedly inhibited cell growth of UM-UC-3 bladder cancer cells [17]. RRM2 upregulation has been demonstrated to increase cell proliferation and the malignant potential of certain types of cancers, and that inhibition of RRM2 reduced cell proliferation in vitro and in vivo [18], [19]. Lin et al. postulated that stable knockdown of the R2 subunit leads to decreased cisplatin-induced gap-filling synthesis in nucleotide excision repair and a reduced dATP level in the G_2_/M phase of the cell cycle, and that this interferes with the repair of single-strand gaps that are otherwise converted into double-strand breaks in the subsequent S phase in HCT-116 human colon cancer cells [20]. Although the RRM2 gene was knocked down and the expression levels of RRM2 were reduced, the SiHa shRRM2 #354 and #962 cell lines still had enough expression levels of RRM2 to reluctantly maintain their cell viabilities after 48 hours culture in the present study (data not showed). Therefore, the cell viabilities of SiHa shRRM2 #354 and #962 cell lines were not reduced to statistical significances, as compared to the control SiHa shLuc cells in our study. The silencing RRM2 alone did not have effect strong enough to reduce cell viability with a statistical significance. The cisplatin binds to DNA and promotes the formation of platinum-DNA adducts, which may establish inter- and intra-strand DNA crosslinks and then inhibits DNA replication [9]. RRM2 did not inhibit cisplatin toxicity specifically. After the addition of cisplatin, the aggravate effect of reduced cell proliferation resulting from inter- and intra-strand DNA crosslinks by cisplatin and from decreased DNA supplement by RRM2 knockdown led to reduced cell viabilities with statistical significances in #354 and #962 cell lines. The silencing of RRM2 expression in SiHa #354 and #962 cell cells augmented cisplatin toxicity.

Our findings showed that the RRM2 immunoreactivity in cancer tissues is higher than that in high-grade dysplasia, low-grade dysplasia and normal tissues of the uterine cervix. RRM2 overexpression has been correlated with cervical carcinogenesis. Fan et al. demonstrated that the R2 protein is not only a rate-limiting component for ribonucleotide reduction but is also capable of cooperating with a variety of oncogenes in the mechanisms of cellular transformation and tumorigenesis [21]. RRM2 subunit overexpression has also been observed in gastric cancer and bladder cancer tumors [17], [22]. In contrast to the findings of Morikawa et al. [17] in which positive staining of carcinoma in situ tissues of the bladder was demonstrated, RRM2 upregulation was not found in the high-grade dysplasia tissues of the uterine cervix in the current study. This may be attributable to the different cooperating oncogenes that transform cells by different mechanisms. Oncogenic stress has been reported to produce dNTP deficiency and consequent DNA replication stress typical of an early oncogenic event [23]. RRM2 has been reported to be transcriptionally regulated by cell cycle-associated factors such as E2F [24]. Furthermore, Liu et al. proposed that an elevated hRRM2 expression causes oxidative stress and activates the Ras/Raf/MAPK signal pathway [25]. The staining of RRM2 was found to be nuclear in this study. Niida and Zhang found that RRM2 is recruited to the nucleus to ensure the local availability of dNTPs for efficient DNA repair synthesis in response to DNA damage [26], [27]. D'Angiolella et al. found that in response to genotoxic stress, RRM2 accumulates in the nuclei of HeLa cervical cancer cells [2]. They further demonstrated that the timing of RRM2 accumulation following DNA damage parallels the timing of DNA repair.

Our study revealed that a positive RRM2 expression is correlated with poor clinicopathological characteristics such as older age, advanced stage other than stage I, deep stromal invasion, large tumors, high grade tumors and positive parametrial invasion. RRM2 tended to be highly expressed in the patients with positive pelvic lymph node metastasis at surgery. In some types of cancer, a high level of RRM2 expression has been reported to be correlated with cellular invasiveness [28], tumour angiogenesis [19], metastasis [25] and poor patient outcomes [29]. Overexpression of RRM2 has been demonstrated to correlate with an increase in cell invasive potential in a human KB oropharyngeal carcinoma cell line [3]. RRM2 may interact with a variety of oncogenes that promote tumor progression and enhance the invasiveness of cancer cells [30]. Morikawa et al. demonstrated that RRM2 overexpression is only significantly associated with muscularis propria invasion, but did not show a significant association with other parameters such as lymph node metastasis [17]. Wang et al. showed that patients with ovarian cancer with clinical FIGO stages III–IV present higher RRM2 gene expressions than those with clinical FIGO stages I–II [31].

In the current study, univariate analysis revealed that cervical cancer patients with deep stromal invasion, large tumor diameter, parametrial invasion, and pelvic lymph node metastasis, as well as positive RRM2 expression have poor mean and median survival, poor 5-year survival rate, and a high HR of death. The patients with positive RRM2 expressions were found to have a significantly lower survival and a higher probability of recurrence. Rosty et al. used an Affymetrix HG-U133A oligonucleotide microarray to show that HPV E6/E7 expression plays a key role in the progression of invasive cervical cancer via the deregulation of cervical cancer proliferation cluster genes with increased average expression levels of 123 unique known genes, including RRM2 [32]. The average expression levels of these genes were higher in cervical cancer tumors with an early relapse than in tumors with a favorable course. RRM2 has also been reported to be a prognostic biomarker in colorectal cancers [33]. Wang et al. found that the survival of patients with a low RRM2 mRNA level is significantly superior to patients with high levels in ovarian cancer [31]. By Cox proportional risk model analysis, the risk of mortality for the patients with high expression levels of RRM2 mRNA was 2.553 times greater than for those with low expressions. However, only deep stromal invasion is an independent prognostic factor for patients with cervical cancer using multivariate analysis in the current study. Many studies have supported that stromal invasion is a powerful independent predictor of overall survival for patient with cervical cancer [34]–[39].

In patients receiving cisplatin-containing treatment, those whose cancer tissues presented a positive RRM2 expression seemed to have worse prognosis than those with a negative RRM2 expression. Cancer cells are more sensitive to the cytotoxic effect of RNR inhibition than normal cells because of the increased need for dNTPs for proliferation and decreased adaptability and low responsiveness to regulatory signals. Thus, this enzyme has long been considered to be an excellent target for cancer chemotherapy [3], [40]. Because the cell cycle-dependent activity of RNR is regulated by the level of RRM2 [8], [21], the role of RRM2 is very important.

There are several limitations to this study. The sample size of the patients who received cisplatin treatment in CCRT or adjuvant chemotherapy protocols was very small. Most of the patients with cervical cancer had stage I or II, and therefore only a few patients received cisplatin-containing treatment. Only 3 patients with negative RRM2 expressions received cisplatin-related adjuvant therapy and they all had better prognosis. This also implies that the cervical cancer patients whose cancer tissues exhibited a negative RRM2 expression had a better prognosis, and therefore the number of these patients who received cisplatin-containing therapy was very limited. In addition, many confounding factors would need to be adjusted, including stromal invasion depth, tumor grade, tumor size, vaginal or parametrial invasion, lymph node metastasis and chemotherapy protocols. We could not therefore analyze the influence of RRM2 expression on the patients who received cisplatin with a statistical significance. Further studies are warranted to define the influence of RRM2 on the exact effect of cisplatin.

To date, few studies have investigated the implication of RRM2 in cervical cancer. Our results suggest that RRM2 could be a new molecular marker for the diagnosis and clinical outcomes of cervical cancer. It is involved in cervical carcinogenesis and predicts poor survival, and it may be a potential therapeutic target including in cisplatin treatment.
